# A retrospective cohort data-linkage study of secondary healthcare use prior to late blood-borne virus diagnosis in Scotland – A missed opportunity for earlier diagnosis?

**DOI:** 10.1017/S0950268826101897

**Published:** 2026-07-23

**Authors:** Maxim Wilkinson, Kirsten Trayner, Karen Maxwell, Beth Cullen, Beth Findlay, Amanda Weir, Robert Hillary, Kirsty Roy, Duncan McCormick, Alison Rodger, Claudia Estcourt, Sharon Hutchinson, Becky Metcalfe

**Affiliations:** 1 School of Health and Life Sciences, Glasgow Caledonian University, UK; 2 Public Health Scotland, UK; 3 UCL, UK; 4 NHS Greater Glasgow and Clyde, UK

**Keywords:** Blood-borne virus transmission, Epidemiology, HIV/AIDS, Hepatitis C, Hepatitis B

## Abstract

The World Health Organization (WHO) has set global goals to eliminate AIDS and viral hepatitis as public health threats. To better target testing, intelligence on missed opportunities for early diagnosis is needed. We determined hospital and emergency department (ED) attendance before late diagnosis of blood-borne viruses (BBVs) in Scotland 5 years to 2 months prior to diagnosis. Late diagnoses were defined as: CD4 count <350 cells/cmm within 90 days of HIV diagnosis; presentation/death from cirrhosis or hepatocellular carcinoma within 1 year of hepatitis C (HCV)/hepatitis B (HBV) diagnosis. Associations were assessed using logistic regression. Among 543 people diagnosed with HIV 37% were late and 2% of people diagnosed with HCV (4,379) and HBV (1,608) were late. Among late diagnoses: 44.1% of HIV, 60.6% of HCV, and 50% of HBV cases previously attended ED and 27.0% of HIV, 46.5% of HCV, and >30% of HBV cases were previously admitted to hospital. The majority were admitted to hospital or attended ED multiple times. Among people newly diagnosed with HIV, those diagnosed late were more likely to have previously attended ED (aOR: 1.41, CI: 0.99–2.02). Further effort is warranted to address the frequent missed opportunities for earlier BBV diagnosis in secondary care.

## Introduction

Blood-borne viruses (BBVs) are a major public health challenge globally. The World Health Organization (WHO) has set goals for eliminating AIDS and viral hepatitis, which include targets for diagnosing 95% of HIV and 90% of viral hepatitis infections by 2030 [1]. Globally, there has been progress towards reaching the HIV targets, with an estimated average of 87% of people living with HIV who know their status [2]. Within Europe, an average of 92% of people living with HIV have been diagnosed across 26 countries [3]. Information on viral hepatitis is comparatively sparse but the available data suggest that the majority of progress has been achieved in high-income countries [4, 5]. Only four European countries provided sufficient data on viral hepatitis, and their progress towards the diagnosis targets is highly variable [3].

In contrast, Scotland has made considerable progress towards the WHO goals [6]: on track to achieve the UNAIDS 95:95:95 targets for HIV (with an estimated 94% diagnosed) [7]; achieved the WHO hepatitis C virus (HCV) 80% treatment target [8]; and achieved WHO targets on hepatitis B virus (HBV) and HCV related mortality and vaccine coverage [9]. However, challenges remain, particularly in identifying those unaware of their infection and ensuring equitable access to testing [6].

Early BBV diagnosis is critical to preventing onward transmission and reducing morbidity and mortality [10]. Understanding previous contact with healthcare is important as these could represent missed opportunities for earlier diagnosis and help inform testing strategies. A missed opportunity in this context is any contact with secondary healthcare during which BBV testing could reasonably have been undertaken. Hospitals and emergency departments (EDs) are promising sites for diagnosing BBVs [11] due to high population usage, and because half of attenders routinely have blood samples taken for testing related to their reason for attendance [12]. While several initiatives have shown hospital admission [13, 14] and ED [15, 16] testing to be effective in detecting undiagnosed BBV infections, the applicability in low-prevalence settings – defined as 0.2% for HIV [17], 0.5% for HCV, and 0.25% for HBV [18] – remains unclear. Scotland represents such a setting, as a country with prevalence rates well below these thresholds in the general population [8–10]. As countries move closer to the WHO elimination targets, developing evidence-based BBV testing strategies tailored to low-prevalence settings will become increasingly important.

This study investigates secondary healthcare use (ED attendance and hospital inpatient admission) in the period prior to a diagnosis among people newly diagnosed with BBVs from 2018 to 2022 in Scotland. Specifically, we aimed to: (i) identify individuals with newly diagnosed BBV infection and classify these as late or non-late diagnoses; (ii) assess their ED attendances and hospital admissions within the 5 years to 2 months before diagnosis to identify potential missed opportunities for testing; and (iii) evaluate any associations between late BBV diagnosis and prior secondary healthcare usage.

## Methods

### Study design and data sources

We conducted a retrospective cohort analysis utilizing linkage of administrative BBV testing and diagnosis data in Scotland (2018–2022), with data on secondary healthcare usage (for people who had been admitted to hospital or attended an ED in Scotland during 2013–2022) to assess secondary healthcare contact prior to diagnosis.

BBV diagnosis data are held by Public Health Scotland (PHS). The HIV diagnosis database includes confirmed HIV cases diagnosed using HIV antigen (Ag)/antibody tests and HIV-1/HIV-2 antibody assays [19]. Additional data on CD4 count, HIV viral load, and immunological data such as avidity are submitted by specialist testing laboratories [8]. The HCV diagnosis database contains epidemiological information for individuals diagnosed by anti-HCV, polymerase chain reaction (PCR), and/or Ag testing [8]. Finally, the HBV testing database contains both positive and negative HBV results for HBV surface antigen (Ag), core immunoglobulin M (IgM), and core antibody [9].

These data were linked to the Scottish Morbidity Records 01 (SMR01) and the Unscheduled Care Datamart using Community Health Index (CHI) numbers, a unique patient identifier number. SMR01 is a national database of hospital admissions and those receiving care in general/acute NHS specialities. Admissions are coded using International Classification of Diseases 10 (ICD-10), and the first code was taken as the primary reason for admission. For each admission, an individual can have multiple episodes relating to different specialties of care. For these cases, the first episode was used to assess reasons for admissions. The Unscheduled Care Datamart contains a record of individuals who have received emergency or urgent care in Scotland. We considered a subset of these records, which included all individuals who had attended ED’s.

### Study population, outcomes, and exposures

The study population comprised all individuals newly diagnosed with a chronic BBV in Scotland between 2018 and 2022, defined as a positive antibody/Ag test for HIV, positive antibody and RNA test for HCV, and positive surface Ag test for HBV. Individuals with missing CHI, or those with a confirmed recent HIV or HBV infection, were excluded. Recent HIV infections were defined by a low immunoglobulin G (IgG) avidity test, while recent HBV infections were defined as an anti-HBc IgM positive result within 2 weeks of diagnosis. Although recent HCV infections could not be confirmed, individuals with self-resolved infection, defined as HCV antibody positive and RNA negative at first diagnosis, were excluded.

The primary outcome of the study was a late BBV diagnosis. For HIV, this was defined as a CD4 count of <350 cells/cmm within 90 days of diagnosis. Individuals without CD4 data were classed as a non-late diagnosis (individuals with late presentation are likely to have CD4 count recorded as diagnosis). For HCV and HBV, late diagnoses were defined as presentation with, or death from, cirrhosis or hepatocellular carcinoma (HCC) within 1 year of being diagnosed.

The primary exposure was attendance at an ED or hospital admission (secondary healthcare) in Scotland that occurred between 5 years and 2 months prior to the BBV diagnosis. Interactions with secondary healthcare use within 2 months prior to BBV diagnosis were excluded, as they may have been related to the diagnosis. A sensitivity analysis also considered admissions/attendances within 2 years and 2 months prior to diagnosis.

Secondary exposures included: age at diagnosis, sex (male/female), health administrative area comprising the two major urban health boards and the rest of Scotland (Greater Glasgow and Clyde (GGC)/Lothian/rest of Scotland), ethnicity (white/other/unknown), diagnosis referral setting (secondary care/sexual health/GP/prison or other/unknown), route of HIV acquisition (gay and bisexual men who have sex with men (GBMSM)/heterosexual sexual intercourse/injecting drug use/other/unknown), and Scottish Index of Multiple Deprivation (SIMD) (1 (most deprived)/2/3–5 (least deprived)).

For those with a secondary healthcare use, covariates included: number of admissions/attendances (1/2–4/5+), clinical speciality (medicine/surgery/other), primary reason for admission (medical/surgical/trauma and orthopaedics), and admitted with HIV indicator condition (yes/no). HIV indicator conditions were identified according to the British HIV Association testing guidelines (Supplementary Table S1) [17]. The presence of HIV indicator conditions across primary and secondary reasons for admissions was assessed. To assess the clinical characteristics of admission, the most recent admission relative to the BBV diagnosis date was considered. Reasons for attendance to ED were not available due to variability in coding.

### Statistical analysis

For each BBV, we compared late diagnosis status (late vs non-late) by demographic and healthcare use covariates using chi-squared or Fisher’s exact tests. We conducted logistic regression models to ascertain if a late diagnosis was associated with usage of secondary healthcare. Separate models were conducted to assess attendance at an ED or admission to hospital and adjusted for age and sex. Our modelling methodology was underpinned by the minimum criteria for sample size considerations in logistic regression, which states that there must be 10 events per independent variable included in the model and covariates to be included in the model were decided *a priori.* To prevent deductive disclosure and due to small numbers in some categories, we have censored values that are less than five.

### Ethics, information governance, and data

This work involved secondary analysis of routinely linked public health surveillance data and was approved by NHS Scotland Public Benefit and Privacy Panel for Health and Social Care (HSC-PBPP) with reference number 2324-0023.The data in this work are not publicly available but access can be requested by applying to the HSC-PBPP.

## Results

### Study cohort

**Figure 1.**
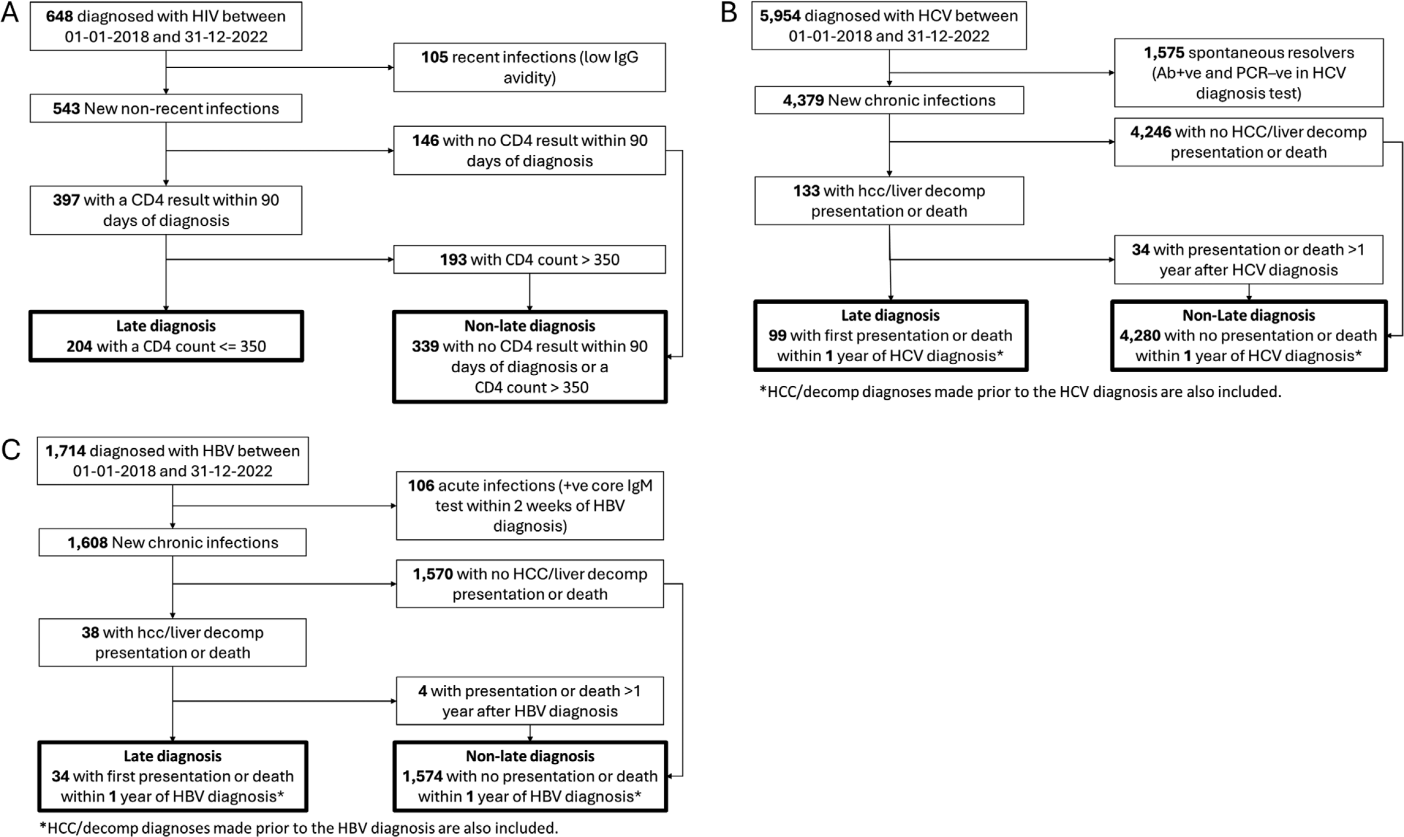
Identification of late diagnoses: (a) HIV; (b) HCV; and (c) HBV. Figure 1. long description.

During the study period, there were 648 new HIV diagnoses in Scotland, of which 105 (16%) were confirmed recent infections based on avidity and excluded. Of the remaining 543 cases, 204 (37%) late HIV diagnoses were identified (Figure 1A). There were 5,954 new HCV antibody positive diagnoses of which 1,575 (26%) who had spontaneously cleared their HCV infection were excluded. This resulted in 4,379 chronic HCV infections among which 99 (2%) late diagnoses were identified (Figure 1B). For HBV, 1,714 diagnoses were made, and 106 (6%) acute infections were excluded based on presence of anti-HBc IgM. Of the remaining 1,608 chronic HBV cases, 34 (2%) were identified as late diagnoses (Figure 1C).

### Demographics of people diagnosed with BBVs in Scotland by late diagnosis

Across all three BBVs, more men than women were diagnosed. We found no differences in late diagnosis by sex for HIV and HCV. However, for HBV, a lower proportion of women (<15%) were diagnosed late when compared to non-late cases (39%) (*p* = 0.001) (Table 1).

**Table 1.** Demographics of individuals diagnosed with blood-borne viruses in Scotland, by late diagnosis, 2018–2022 Table 1. long description.

	HIV				HCV				HBV			
	Total (%col)	Late diagnosis		*p*-value^a^	Total (%col)	Late diagnosis		*p*-value^a^	Total (%col)	Late diagnosis		*p*-value^a^
		Yes (%col)	No (%col)			Yes (%col)	No (%col)			Yes (%col)	No (%col)	
**Total number diagnoses**	543	204	339	-	4,379	99	4,280	-	1,608	34	1,574	-
**Age at diagnosis (mean)^b^ (median)**	40 (38)	42 (41)	39 (36)		41 (40)	56 (56)	41 (40)	-	39 (37)	58 (60)	39 (37)	-
**Gender**												
Female	127 (24.3%)	45 (22.1%)	82 (24.2%)		1,348 (30.8%)	25 (25.3%)	1,323 (30.9%)		614 (38.2%)	<5 (<15.0%)	610 (38.8%)	
Male	416 (76.6%)	159 (77.9%)	257 (75.8%)	0.643	3,002 (68.6%)	74 (74.7%)	2,928 (68.4%)	0.255	>980 (>60%)	>29 (>85.0%)	>950 (>60%)	0.001
Unknown	-	-	-		29 (0.7%)	-	29 (0.7%)		<5 (<1%)	-	<5 (<1%)	
**Year of diagnosis**												
2018	159 (29.3%)	60 (29.4%)	99 (29.2%)		1,087 (24.8%)	21 (21.2%)	1,066 (24.9%)		325 (20.2%)	8 (23.5%)	317 (20.1%)	
2019	118 (21.7%)	47 (23.0%)	71 (20.9%)		1,082 (24.7%)	25 (25.3%)	1,057 (24.7%)		373 (23.2%)	5 (14.7%)	368 (23.4%)	
2020	94 (17.3%)	32 (15.7%)	62 (18.3%)		692 (15.8%)	16 (16.2%)	676 (15.8%)		228 (14.2%)	7 (20.6%)	221 (14.0%)	
2021	76 (14.0%)	27 (13.2%)	49 (14.5%)		785 (17.9%)	17 (17.9%)	768 (17.9%)		286 (17.8%)	8 (23.5%)	278 (17.7%)	
2022	96 (17.7%)	38 (18.6%)	58 (17.1%)	0.901	733 (16.7%)	20 (20.2%)	713 (16.7%)	0.860	396 (24.6%)	6 (17.6%)	390 (24.8%)	0.462
**Health board**												
Greater Glasgow and Clyde	190 (35.0%)	82 (40.2%)	108 (31.9%)		1,133 (25.9%)	26 (26.3%)	1,107 (25.9%)		544 (33.8%)	15 (44.1%)	529 (33.6%)	
Lothian	77 (14.2%)	31 (15.2%)	46 (13.6%)		585 (13.4%)	10 (10.1%)	575 (13.4%)		322 (20.0%)	5 (14.7%)	317 (20.1%)	
Rest of Scotland	276 (50.8%)	91 (44.6%)	185 (54.6%)	0.072	2,661 (60.8%)	63 (63.6%)	2,598 (60.7%)	0.662	742 (46.1%)	14 (41.2%)	728 (46.3%)	0.414
**Ethnicity^b^**												
White	364 (67.0%)	132 (64.7%)	232 (68.4%)		3,423 (78.2%)	79 (79.8%)	3,344 (78.1%)		377 (23.4%)	17 (50.0%)	360 (22.9%)	
Other	139 (25.6%)	61 (29.9%)	78 (23.0%)		210 (4.8%)	10 (10.1%)	200 (4.7%)		816 (50.7%)	>10 (>40.0%)	802 (51.0%)	
Unknown	40 (7.4%)	11 (5.4%)	29 (8.6%)	0.141	746 (17.0%)	10 (10.1%)	736 (17.2%)	0.035	415 (25.8%)	>5 (<10.0%)	412 (26.2%)	0.008
**Referral setting**												
Secondary care	307 (56.5%)	131 (64.2%)	176 (51.9%)		1,765 (40.3%)	66 (66.7%)	1,699 (39.7%)		573 (35.6%)	22 (64.7%)	551 (35.0%)	
Sexual Health	133 (24.5%)	39 (19.1%)	94 (27.7%)		172 (3.9%)	0 (0.0%)	172 (4.0%)		98 (6.1%)	0 (0.0%)	98 (6.2%)	
GP	52 (9.6%)	21 (10.3%)	31 (9.1%)		838 (19.1%)	14 (14.1%)	824 (19.3%)		635 (39.5%)	7 (20.6%)	628 (39.9%)	
Prison/Other/Unknown	51 (9.4%)	13 (6.4%)	38 (11.2%)	0.014	1,604 (36.6%)	19 (19.2%)	1,585 (37.0%)	< 0.001	302 (18.8%)	5 (14.7%)	297 (18.9%)	0.004
**Scottish index of multiple deprivation^b^**												
1 (most deprived)	191 (32.5%)	75 (36.8%)	116 (34.2%)		1,906 (43.5%)	45 (45.5%)	1,861 (43.5%)		539 (33.5%)	10 (29.4%)	529 (33.6%)	
2	89 (16.4%)	34 (16.7%)	55 (16.2%)		999 (22.8%)	23 (23.2%)	976 (22.8%)		305 (19.0%)	11 (32.4%)	294 (18.7%)	
3–5 (least deprived)	194 (35.7%)	84 (41.2%)	110 (32.4%)		1,020 (23.3%)	31 (31.3%)	989 (23.1%)		623 (38.7%)	13 (38.2%)	610 (38.8%)	
Unknown	69 (12.7%)	11 (5.4%)	58 (17.1%)	0.626	454 (10.4%)	-	454 (10.6%)	0.471	141 (8.8%)	-	141 (9.0%)	0.235
**Route of acquisition^b^**												
GBMSM	148 (43.7%)	82 (40.2%)	148 (43.7%)		-	-	-	-	-	-	-	-
Heterosexual sexual intercourse	121 (35.7%)	92 (45.1%)	121 (35.7%)		-	-	-	-	-	-	-	-
Injecting drug use	39 (11.5%)	10 (4.9%)	39 (11.5%)		-	-	-	-	-	-	-	-
Other/Unknown	31 (9.1%)	20 (9.8%)	31 (9.1%)	0.023	-	-	-	-	-	-	-	-

a Generated from chi-squared or Fisher’s test.

b Unknown values have been excluded from test.

From 2018 to 2022, HIV diagnosis declined for both late (60 to 38 cases) and non-late diagnoses (99 to 58). HCV late diagnoses remained stable (21 to 20) with a decreasing trend for HCV non-late diagnoses (1,087 to 733 cases). HBV diagnoses increased from 325 in 2018 to 396 in 2022 with no differences observed between late and non-late diagnoses (Table 1).

More diagnoses were made in NHS GGC than any other health board for all three BBVs (35.0%, 25.9%, and 33.8% for HIV, HCV, and HBV, respectively). For NHS GGC, NHS Lothian, and the combined rest of Scotland, there were minimal differences between proportion of late and non-late diagnoses for any of the BBVs. For HIV and HCV, the majority of cases were of white ethnicity (67.0% and 78.2%, respectively) and there were no differences in ethnicity between late and non-late diagnoses. Differences were observed for HBV where 50.0% of late diagnoses were classed as being of white ethnicity compared to 22.9% of non-late diagnoses (*p* = 0.008) (Table 1).

For HIV, the majority of both late and non-late infections were diagnosed in secondary care (64.2% and 51.9%, respectively). Differences were observed for HCV (*p* < 0.001) and HBV (*p* = 0.004) where most late diagnoses were made in secondary care (66.7% and 64.7%, respectively). The percentage of non-late diagnoses for HCV was similar for secondary care and prison/other/unknown referral settings (39.7% and 37.0%, respectively), while for HBV, 39.9% of diagnoses were made in GP surgeries.

Among people diagnosed with HIV, significant differences in route of acquisition were observed between the late and non-late diagnoses (*p* = 0.023). HIV acquired via heterosexual sexual intercourse was the most common route of transmission among those with late HIV diagnoses (45.1%) while GBMSM individuals made up a greater proportion of the non-late diagnoses (43.7%). Injecting drug use was more frequently reported among non-late HIV diagnoses compared to late diagnoses (11.5% vs 4.9%, respectively; *p* = 0.004) (Table 1).

The majority of cases for all three BBVs were in the two lowest SIMD quintiles, which comprise individuals from areas with the highest deprivation, (HIV: 48.9%, HCV: 66.3%, and HBV: 52.5%), with similar findings across late and non-late diagnoses.

### Secondary healthcare interactions

#### Attendance at emergency departments

A higher proportion of individuals attended an ED in the period between 5 years and 2 months prior to a diagnosis for HCV (65.0%, 2,848) than for HIV (39.6%, 215) or HBV (23.2%, 373) (Table 2); this trend was similarly observed for the two-year period prior to diagnosis (Supplementary Table S2). A higher proportion of people with late HBV diagnosis had attended ED than those diagnosed non-late (50.0% and 22.6%, respectively; *p* < 0.001). No significant difference in ED attendance was observed between those diagnosed late and non-late for HIV (44.1% and 36.9%, respectively) and HCV (60.6% and 65.1%, respectively). For those who attended ED in the period 2 months to 5 years prior to diagnosis, the majority attended more than once (57.2%, 77.7%, and 54.4% for HIV, HCV, and HBV, respectively) (Table 2).

**Table 2.** Usage of secondary healthcare in the period between 5 years and 2 months prior to diagnosis for individuals newly diagnosed with blood-borne viruses in Scotland, by late diagnosis, 2018–2022 Table 2. long description.

	HIV				HCV				HBV			
	Total (%col)	Late diagnosis		*p*-value^a^	Total (%col)	Late diagnosis		*p*-value^a^	Total (%col)	Late diagnosis		*p*-value^a^
		Yes (%col)	No (%col)			Yes (%col)	No (%col)			Yes (%col)	No (%col)	
**Total number of diagnoses**	543	204	339		4,379	99	4,280		1,608	34	1,574	
**A) Emergency departments (ED)**												
**Attended ED**												
Yes	215 (39.6%)	90 (44.1%)	125 (36.9%)		2,848 (65.0%)	60 (60.6%)	2,788 (65.1%)		373 (23.2%)	17 (50.0%)	356 (22.6%)	
No	328 (60.4%)	114 (55.9%)	214 (63.1%)	0.113	1,531 (35.0%)	39 (39.4%)	1,492 (34.9%)	0.407	1,235 (76.8%)	17 (50.0%)	1,218 (77.4%)	< 0.001
**Number of ED attendances^b^**												
Once	92 (42.8%)	39 (43.3%)	53 (42.4%)		633 (22.2%)	20 (33.3%)	613 (22.0%)		170 (45.6%)	8 (47.1%)	162 (45.5%)	
2 to 4	83 (38.6%)	40 (44.4%)	43 (34.4%)		1,086 (38.1%)	25 (41.7%)	1,061 (38.1%)		144 (38.6%)	>5 (>15.0%)	138 (38.8%)	
5+	40 (18.6%)	11 (12.2%)	29 (23.2%)	0.092	1,129 (39.6%)	15 (25.0%)	1,114 (40.0%)	0.031	59 (15.8%)	<5 (<15.0%)	56 (15.7%)	0.956
**Median (IQR) number of attendances^b^**	2 (1–3)	2 (1–3)	2 (1–4)	-	3 (2–7)	2.5 (1–4)	3 (2–7)		2 (1–3)	2 (1–3)	2 (1–4)	
**B) Secondary care**												
**Hospital admission**												
Yes	137 (25.2%)	55 (27.0%)	82 (24.2%)		2,091 (47.8%)	46 (46.5%)	2,045 (47.8%)		240 (14.9%)	13 (38.2%)	227 (14.4%)	
No	406 (74.8%)	149 (73.0%)	257 (75.8%)	0.534	2,288 (52.2%)	53 (53.5%)	2,235 (52.2%)	0.875	1,368 (85.1%)	21 (61.8%)	1,347 (85.6%)	< 0.001
Number of hospital admissions b												
Once	60 (43.6%)	24 (43.6%)	36 (43.9%)		812 (38.8%)	20 (43.5%)	792 (38.7%)		102 (42.5%)	>5 (>30.0%)	98 (43.2%)	
2 to 4	56 (40.9%)	26 (47.3%)	30 (36.6%)		893 (42.7%)	18 (39.1%)	875 (42.8%)		87 (36.2%)	>5 (<50.0%)	81 (35.7%)	
5+	21 (15.3%)	5 (9.1%)	16 (19.5%)	0.196	386 (18.5%)	8 (17.4%)	378 (18.5%)	0.806	51 (21.2%)	<5 (<20.0%)	48 (21.1%)	0.666
**Median (IQR) number of admissions^b^**	2 (1–3)	2 (1–3)	1 (1–4)	-	2 (1–4)	2 (1–4)	2 (1–4)		2 (1–4)	2 (1–4)	2 (1–4)	
Primary reason for admission c												
Medical	89 (65.0%)	29 (52.7%)	60 (73.2%)		1,318 (63.0%)	25 (54.3%)	1,293 (63.2%)		140 (58.3%)	10 (76.9%)	130 (57.3%)	
Surgical	31 (22.6%)	20 (36.4%)	11 (13.4%)		356 (17.0%)	11 (23.9%)	345 (16.9%)		80 (33.3%)	<5 (>5.0%)	79 (34.8%)	
Trauma and orthopaedics	17 (12.4%)	6 (10.9%)	11 (13.4%)	<0.001	417 (19.9%)	10 (21.7%)	407 (19.9%)	0.377	20 (8.3%)	>5 (>15.0%)	18 (7.9%)	0.058
**Admitted with HIV indicator condition (primary/secondary)^b^** ^,^ **^c^**												
Yes	24 (17.5%)	10 (18.2%)	14 (17.1%)			-	-	-		-	-	-
No	113 (82.5%)	45 (81.8%)	68 (82.9%)	1.000		-	-	-		-	-	-

a Generated from chi-squared or Fisher’s test.

b Among people who were admitted to hospital or attended ED.

c Relates to most recent admission prior to diagnosis.

#### Admission to hospital

A lower proportion of people with a BBV diagnosis were admitted to hospital between 5 years and 2 months prior to a diagnosis than attended an ED. Overall, 25.2% (137) of HIV, 47.8% (2,091) of HCV, and 14.9% (240) of HBV diagnoses had at least one attendance. Similar to findings for ED attendance, there was no difference in hospital admission between those diagnosed late and non-late for HIV and HCV, but a higher proportion of those diagnosed late with HBV had been admitted to hospital compared to non-late (38.2% and 14.4%, respectively; *p* < 0.001). Of those who were admitted to hospital, the majority had more than one admission for all three BBVs (56.4%, 61.2%, and 57.5% for HIV, HCV, and HBV, respectively) (Table 2).

ICD-10 coding showed the most recent admission prior to diagnosis was due to a medical diagnosis across HIV (65.0%), HCV (63.0%), and HBV (58.3%). A higher proportion of people with a late diagnosis were admitted to hospital for surgical related reasons compared to non-late diagnoses for HIV (36.4% and 13.4%; *p* < 0.001) and medical reasons for HBV (76.9% and 57.3%, respectively; *p* = 0.058); there were no difference in the types of hospital admission between late and non-late diagnoses for HCV (Table 2).

Among people diagnosed with HIV and admitted to hospital, a similar proportion of late (18.2%) and non-late (17.1%) diagnoses had an HIV indicator condition documented as a primary or secondary condition during their most recent admission prior to diagnosis (Table 2). Among all (non-recent) HIV diagnoses, 24 (4.4% of 543) were admitted to hospital with an HIV indicator condition listed as the primary reason for admission in the period between 2 months and 5 years prior to diagnosis.

### Associations between late BBV diagnosis and secondary healthcare use

In regression analysis adjusting for age and sex, ED attendance in the period between 2 months and 5 years prior to diagnosis was associated with late (compared to non-late) diagnosis for HIV (adjusted odds ratio (aOR): 1.41, confidence interval (CI): 0.99–2.02) but not for HCV (aOR: 1.27, CI: 0.82–1.97) or HBV (aOR: 1.68, CI: 0.79–3.51) (Table 3). A sensitivity analysis considering a two-year period prior to diagnosis yielded similar results to the five-year period for all three BBVs (Supplementary Table S3).

**Table 3.** Associations between attending an emergency department in the period between 5 years and 2 months prior to diagnosis and late diagnosis for individuals newly diagnosed with BBVs in Scotland, 2018–2022 Table 3. long description.

	N	Late diagnosis (% of N)	Univariate		Multivariate^a^	
			OR (95% CI)	*p*-value	aOR (95% CI)	*p*-value
**HIV**						
**ED attendance**						
No	328	114 (34.8%)	1		1	
Yes	215	90 (41.9%)	1.35 (0.94, 1.93)	0.095	1.41 (0.99, 2.02)	0.058
**HCV**						
**ED attendance**						
No	1,502	39 (2.6%)	1		1	
Yes	2,848	60 (2.1%)	0.81 (0.54, 1.22)	0.304	1.27 (0.82, 1.97)	0.288
**HBV**						
**ED attendance**						
No	1,231	17 (1.38%)	1		1	
Yes	373	17 (4.56%)	3.41 (1.71, 6.79)	< 0.001	1.68 (0.79, 3.51)	0.169

a Adjusted for age and sex.

There was no association between hospital admission in the period between 2 months and 5 years prior to a diagnosis and a late diagnosis for HIV (aOR: 1.08, CI: 0.72–1.61), HCV (aOR: 1.11, CI: 0.73–1.68), or HBV (aOR: 1.13, CI: 0.48–2.56) compared to not being admitted (Table 4). In a sensitivity analysis, no associations were found in adjusted regression analyses considering hospital admission in the period between 2 months and 2 years prior to diagnosis (Supplementary Table S4).

**Table 4.** Associations between admission to hospital in the period between 5 years and 2 months prior to diagnosis and late diagnosis for individuals newly diagnosed with BBVs in Scotland, 2018–2022 Table 4. long description.

	N	Late diagnosis (% of N)	Univariate		Multivariate^a^	
			OR (95% CI)	*p*-value	aOR (95% CI)	*p*-value
**HIV**						
**Admitted to hospital**						
No	406	149 (36.7%)	1		1	
Yes	137	55 (40.1%)	1.16 (0.77, 1.72)	0.472	1.08 (0.72, 1.61)	0.715
**HCV**						
**Admitted to hospital**						
No	2,259	53 (2.4%)	1		1	
Yes	2,091	46 (2.2%)	0.94 (0.63, 1.4)	0.747	1.11 (0.73, 1.68)	0.617
**HBV**						
**Admitted to hospital**						
No	1,364	21 (1.5%)	1		1	
Yes	240	13 (5.4%)	3.66 (1.76, 7.33)	< 0.001	1.13 (0.48, 2.56)	0.775

a Adjusted for age and sex.

## Discussion

This is the first national study to conduct a retrospective analysis of both ED attendance and admission to hospital prior to a late diagnosis for three BBVs (HIV, HCV, and HBV), providing evidence to address gaps in current practice, and inform the development of new BBV testing interventions in low-prevalence settings. We have shown an appreciable proportion of those diagnosed with a BBV had attended an ED or been admitted to hospital prior to their first diagnosis, often multiple times, highlighting potentially missed testing opportunities. Furthermore, ED attendance was associated with late diagnosis of HIV indicating that individuals with late-stage disease are more likely to present to emergency services than those with an earlier stage of disease; thus, ED represents an important setting to diagnose individuals who are at risk of life threatening complications and with an urgent need to engage in clinical care and treatment.

This work highlights the variation in BBV transmission risk and diagnostic pathways. HIV infection is predominantly transmitted in Scotland through sexual contact [20] in contrast to HCV where transmission primarily occurs through injecting drug use [21]. HBV is transmitted through a range of routes [22], but in Scotland, it is mainly associated with individuals who have immigrated from higher-prevalence countries. These differences in transmission routes likely impact the variation in the setting for diagnosis. For individuals with non-late diagnoses, many were diagnosed in secondary care settings, particularly for HIV and to a lesser extent for HCV and HBV; HCV was also often diagnosed in prisons or other settings such as drug services, and HBV was primarily diagnosed in GP surgeries. For those diagnosed late with a BBV, secondary healthcare was the dominant setting of referral for all three BBVs. This could be due to several reasons, including the need to present to secondary care as a consequence of symptoms associated with their advancing stage of disease or because people are not engaged with a range of other community-based services (including sexual health, drug services, and prisons) where BBV testing is routinely undertaken.

Despite the significant testing efforts already taking place in secondary healthcare settings, we observed frequent missed opportunities to diagnose people. HIV indicator testing is recommended in national guidelines to improve early diagnosis [18], but fewer than 5% of all individuals diagnosed in our study had an indicator condition recorded as a primary or secondary reason for hospital admission in the preceding 5 years. This is similar to previous work in Australia [23] and suggests that HIV indicator-condition-based testing may be insufficient in secondary care settings to reach those with undiagnosed infection, and that broader testing approaches may be required to address these missed opportunities.

However, implementing opt-out BBV testing in all secondary care settings would have significant resource implications, and our analysis demonstrates more frequent ED attendances than hospital admissions among secondary healthcare users. This was particularly the case for HCV, highlighting how EDs could be important for diagnoses among hard-to-reach populations such as people who inject drugs. Additionally, data from a recent ED opt-out programme in England identified that 63% of eligible attendees had no evidence of a previous BBV test [16]. Opt-out testing in the ED can also identify previously diagnosed BBV infections offering the potential to re-engage in care where needed. Our results suggest that, despite Scotland being a low-prevalence setting for all three BBVs [8–10, 18], ED testing could help to identify undiagnosed infections.

It is well known that EDs are important settings for identifying individuals with undiagnosed BBV infections, particularly in high-prevalence settings [23–25]. Despite this, the extent of missed opportunities for earlier HIV diagnosis in ED varies dramatically, even between geographically proximal hospitals [26]. Internationally, regional and national-level studies have reported that between 16% and 60% of individuals subsequently diagnosed with a BBV had previously attended an ED without being tested [16, 27–29]. The high variation may arise from diverse cohort definitions; most studies considered all individuals diagnosed with a BBV and lacked a focus on those diagnosed late with their infection. Our study contributes to this evidence base by providing, to our knowledge, the first national data-linkage analysis spanning three BBVs. By focusing on individuals diagnosed late, we aimed to ensure that missed opportunities reflected genuine occasions when infections were already present but testing did not occur.

Our study has several limitations. Firstly, we have focussed on secondary care as an environment for testing, but there are many other healthcare settings that people who are at higher risk of BBV acquisition interact with. Prisons and drug services have been the subject of separate studies which have informed the introduction of Scottish Government BBV testing targets in these settings [8]. Further attention is warranted to the role of primary care, in the context of the key role GPs already play, in diagnosing BBV, particularly HBV, infection. Secondly, the definition of late diagnosis of HBV and HCV in this study was based on available data on severe liver disease. As such, we focussed on ‘very late’ diagnoses and thus do not consider a broader definition of late diagnosis including those who had developed cirrhosis. Thirdly, blood samples are only routinely collected in approximately half of people attending ED, but no specific information on this was available in our data to assess whether there was a missed opportunity for diagnosis based on an opt-out testing approach. Finally, when considering the associations between a late BBV diagnosis and secondary care interactions, we were limited to adjusting for age and sex. Data on risk were only available for HIV, and the number of late diagnoses for each BBV, particularly HBV, was small, increasing the risk of overinterpreting the statistical analyses performed as more covariates are added.

## Conclusion

This study presents an approach for countries to assess the extent of missed opportunities for earlier BBV diagnosis in secondary care. This information is essential for evaluating and informing testing strategies, including in countries such as Scotland where BBV prevalence is low and elimination targets are close to being achieved. Our findings show that many individuals diagnosed with a BBV at a later stage had previously engaged with secondary care – particularly through ED attendances and to a lesser extent hospital admissions – prior to their diagnosis. This indicates that opportunities for earlier diagnoses still exist, even in near-eliminations settings and underscores the potential of secondary care, particularly EDs as an underutilized site for BBV detection. Thus, alternative testing strategies in secondary care, such as an opt-out model in EDs, should be considered to reduce missed opportunities for earlier diagnosis.

## Supporting information

10.1017/S0950268826101897.sm001 Wilkinson et al. supplementary material Wilkinson et al. supplementary material

## Data Availability

The data that support these findings are not openly available. They contain sensitive healthcare information that includes information about the diagnosis of HIV and viral hepatitis. People who have been diagnosed with blood-borne viruses often experience stigma, and as such, it is important to avoid potential disclosure. While the data are pseudonymized for analysis, there is still the chance of disclosure due to the small numbers involved in some of the groups. We have added a data availability statement to the manuscript detailing how interested parties would be able to access the data.

## Supplementary material

The supplementary material for this article can be found at http://doi.org/10.1017/S0950268826101897.

## Long descriptions

### Figure 1 Long description

Panel A: H I V flowchart. Starts with 648 diagnosed. 105 recent infections are excluded. Of 543 new non-recent infections, 146 had no C D 4 result within 90 days and 397 had a result. Of those with a result, 193 had a C D 4 count greater than 350. Final outcomes: Late diagnosis includes 204 with C D 4 count less than or equal to 350. Non-late diagnosis includes 339 with no C D 4 result or a count greater than 350.

Panel B: H C V flowchart. Starts with 5,954 diagnosed. 1,575 spontaneous resolvers are excluded. Of 4,379 new chronic infections, 4,246 had no H C C or liver decompensation presentation or death, while 133 had such a presentation. Of those 133, 34 occurred more than 1 year after diagnosis. Final outcomes: Late diagnosis includes 99 with first presentation or death within 1 year. Non-late diagnosis includes 4,280 with no presentation or death within 1 year.

Panel C: H B V flowchart. Starts with 1,714 diagnosed. 106 acute infections are excluded. Of 1,608 new chronic infections, 1,570 had no H C C or liver decompensation presentation or death, while 38 had such a presentation. Of those 38, 4 occurred more than 1 year after diagnosis. Final outcomes: Late diagnosis includes 34 with first presentation or death within 1 year. Non-late diagnosis includes 1,574 with no presentation or death within 1 year.

Navigate back to Figure 1.

### Table 1 Long description

The table presents demographic data for H I V, H C V, and H B V.

Total number of diagnoses:

* H I V: 543 total, 204 late, 339 not late.

* H C V: 4,379 total, 99 late, 4,280 not late.

* H B V: 1,608 total, 34 late, 1,574 not late.

Age at diagnosis (mean and median):

* H I V: Total 40 (38), Late 42 (41), Not late 39 (36).

* H C V: Total 41 (40), Late 56 (56), Not late 41 (40).

* H B V: Total 39 (37), Late 58 (60), Not late 39 (37).

Gender distribution:

* H I V: 76.6 percent male, 24.3 percent female.

* H C V: 68.6 percent male, 30.8 percent female.

* H B V: Over 60 percent male, 38.2 percent female.

Year of diagnosis (2018 to 2022):

* H I V: Decreased from 29.3 percent in 2018 to 17.7 percent in 2022.

* H C V: Decreased from 24.8 percent in 2018 to 16.7 percent in 2022.

* H B V: Fluctuated between 20.2 percent in 2018 and 24.6 percent in 2022.

Health board:

* Greater Glasgow and Clyde accounts for 35.0 percent of H I V, 25.9 percent of H C V, and 33.8 percent of H B V cases.

Ethnicity:

* White: 67.0 percent of H I V, 78.2 percent of H C V, 23.4 percent of H B V.

* Other: 25.6 percent of H I V, 4.8 percent of H C V, 50.7 percent of H B V.

Referral setting:

* Secondary care is the primary setting for H I V (56.5 percent) and H C V (40.3 percent), while G P is the primary setting for H B V (39.5 percent).

Scottish index of multiple deprivation:

* Most deprived (1) accounts for 32.5 percent of H I V, 43.5 percent of H C V, and 33.5 percent of H B V.

Route of acquisition (H I V only):

* G B M S M: 43.7 percent.

* Heterosexual sexual intercourse: 35.7 percent.

* Injecting drug use: 11.5 percent.

Navigate back to Table 1.

### Table 2 Long description

The table is divided into two main sections: A) Emergency departments (E D) and B) Secondary care. It compares healthcare usage between 5 years and 2 months prior to diagnosis for H I V, H C V, and H B V.

Section A: Emergency departments (E D)

* H I V: 543 total diagnoses. 39.6% attended E D. Late diagnosis (Yes) was 44.1% versus 36.9% for No (p-value 0.113). Median attendances were 2.

* H C V: 4,379 total diagnoses. 65.0% attended E D. Late diagnosis (Yes) was 60.6% versus 65.1% for No (p-value 0.407). Median attendances were 3.

* H B V: 1,608 total diagnoses. 23.2% attended E D. Late diagnosis (Yes) was 50.0% versus 22.6% for No (p-value < 0.001). Median attendances were 2.

Section B: Secondary care (Hospital admission)

* H I V: 25.2% admitted. Late diagnosis (Yes) was 27.0% versus 24.2% for No (p-value 0.534). Primary reason for admission was Medical (65.0%). 17.5% were admitted with an H I V indicator condition.

* H C V: 47.8% admitted. Late diagnosis (Yes) was 46.5% versus 47.8% for No (p-value 0.875). Primary reason for admission was Medical (63.0%).

* H B V: 14.9% admitted. Late diagnosis (Yes) was 38.2% versus 14.4% for No (p-value < 0.001). Primary reason for admission was Medical (58.3%).

Median number of admissions for all three viruses was 2, with an interquartile range typically between 1 and 4.

Navigate back to Table 2.

### Table 3 Long description

The table is divided into three sections for H I V, H C V, and H B V. Columns include N, Late diagnosis (percentage of N), Univariate O R (95 percent C I), Univariate p-value, Multivariate a O R (95 percent C I), and Multivariate p-value.

* H I V section: For No E D attendance (N equals 328), late diagnosis is 114 (34.8 percent). For Yes E D attendance (N equals 215), late diagnosis is 90 (41.9 percent). Multivariate a O R is 1.41 (0.99, 2.02) with a p-value of 0.058.

* H C V section: For No E D attendance (N equals 1,502), late diagnosis is 39 (2.6 percent). For Yes E D attendance (N equals 2,848), late diagnosis is 60 (2.1 percent). Multivariate a O R is 1.27 (0.82, 1.97) with a p-value of 0.288.

* H B V section: For No E D attendance (N equals 1,231), late diagnosis is 17 (1.38 percent). For Yes E D attendance (N equals 373), late diagnosis is 17 (4.56 percent). Univariate O R is 3.41 (1.71, 6.79) with a p-value less than 0.001. Multivariate a O R is 1.68 (0.79, 3.51) with a p-value of 0.169.

Multivariate analysis is adjusted for age and sex.

Navigate back to Table 3.

### Table 4 Long description

The table is divided into three sections for H I V, H C V, and H B V. Each section compares individuals who were admitted to the hospital versus those who were not.

* H I V section:

- No admission: N equals 406, late diagnosis 149 (36.7 percent), O R and a O R are 1.

- Yes admission: N equals 137, late diagnosis 55 (40.1 percent), univariate O R 1.16 (95 percent C I 0.77 to 1.72, p-value 0.472), multivariate a O R 1.08 (95 percent C I 0.72 to 1.61, p-value 0.715).

* H C V section:

- No admission: N equals 2,259, late diagnosis 53 (2.4 percent), O R and a O R are 1.

- Yes admission: N equals 2,091, late diagnosis 46 (2.2 percent), univariate O R 0.94 (95 percent C I 0.63 to 1.4, p-value 0.747), multivariate a O R 1.11 (95 percent C I 0.73 to 1.68, p-value 0.617).

* H B V section:

- No admission: N equals 1,364, late diagnosis 21 (1.5 percent), O R and a O R are 1.

- Yes admission: N equals 240, late diagnosis 13 (5.4 percent), univariate O R 3.66 (95 percent C I 1.76 to 7.33, p-value less than 0.001), multivariate a O R 1.13 (95 percent C I 0.48 to 2.56, p-value 0.775).

Multivariate analysis is adjusted for age and sex.

Navigate back to Table 4.
