# Supplementary material for: A retrospective cohort data-linkage study of secondary healthcare use prior to late blood-borne virus diagnosis in Scotland – A missed opportunity for earlier diagnosis?
Source: Epidemiol Infect. 2026 Jul 23;154:e109. doi: 10.1017/S0950268826101897 (PMC13459007; doi:10.1017/S0950268826101897)
Supplement: Supplementary file 1 [file S0950268826101897sup001.docx]

# Supplementary materials

Table S1 – ICD 10 codes utilised to identify HIV indicator conditions within hospital admissions.

| **ICD 10 code** | **Description** |
| --- | --- |
| C21 | Malignant neoplasm of anus and anal canal |
| C34 | Malignant neoplasm of bronchus and lung |
| C46 | Kaposi sarcoma |
| C53 | Malignant neoplasm of cervix uteri |
| C83 | Non-follicular lymphoma |
| C85 | Other and unspecified types of non-Hodgkin lymphoma |
| C86 | Other specified types of T/NK-cell lymphoma |
| C88 | Malignant immunoproliferative diseases |
| D15 | Benign neoplasm of other and unspecified intrathoracic organs |
| D16 | Benign neoplasm of bone and articular cartilage |
| N87 | Dysplasia of cervix uteri |
| A02 | Other salmonella infections |
| A15 | Respiratory tuberculosis, bacteriologically and histologically confirmed |
| A16 | Respiratory tuberculosis, not confirmed bacteriologically or histologically |
| A17 | Tuberculosis of nervous system |
| A18 | Tuberculosis of other organs |
| A19 | Miliary tuberculosis |
| A31 | Infection due to other mycobacteria |
| A60 | Anogenital herpesviral [herpes simplex] infection |
| A81 | Atypical virus infections of central nervous system |
| A87 | Viral meningitis |
| B02 | Zoster [herpes zoster] |
| B15 | Acute hepatitis A |
| B19 | Unspecified viral hepatitis |
| B18 | Chronic viral hepatitis |
| B21 | Human immunodeficiency virus [HIV] disease resulting in malignant neoplasms |
| B25 | Cytomegaloviral disease |
| B17 | Other acute viral hepatitis |
| B55 | Leishmaniasis |
| A07 | Other protozoal intestinal diseases |
| B57 | Chagas disease |
| B58 | Toxoplasmosis |
| B37 | Candidiasis |
| B39 | Histoplasmosis |
| B45 | Cryptococcosis |
| B38 | Coccidioidomycosis |
| B48 | Other mycoses, not elsewhere classified |
| B09 | Unspecified viral infection characterized by skin and mucous membrane lesions |
| B27 | Infectious mononucleosis |
| K13 | Other diseases of lip and oral mucosa |
| A51 | Early syphilis |
| A52 | Late syphilis |
| A53 | Other and unspecified syphilis |
| A54 | Gonococcal infection |
| A55 | Chlamydial lymphogranuloma (venereum) |
| A56 | Other sexually transmitted chlamydial diseases |
| A57 | Chancroid |
| A58 | Granuloma inguinale |
| A59 | Trichomoniasis |
| A63 | Other predominantly sexually transmitted diseases, not elsewhere classified |
| A64 | Unspecified sexually transmitted disease |
| A81 | Atypical virus infections of central nervous system |
| J13 | Pneumonia due to Streptococcus pneumoniae |
| J15 | Bacterial pneumonia, not elsewhere classified |
| J16 | Pneumonia due to other infectious organisms, not elsewhere classified |
| J18 | Pneumonia, organism unspecified |
| D72 | Other disorders of white blood cells |
| L21 | Seborrhoeic dermatitis |
| L40 | Psoriasis |
| N15 | Other renal tubulo-interstitial diseases |
| N18 | Chronic kidney disease |
| G35 | Multiple sclerosis |
| G56 | Mononeuropathies of upper limb |
| G57 | Mononeuropathies of lower limb |
| G58 | Other mononeuropathies |
| G59 | Mononeuropathy in diseases classified elsewhere |
| G61 | Inflammatory polyneuropathy |
| G90 | Disorders of autonomic nervous system |
| K52 | Other noninfective gastroenteritis and colitis |
| R50 | Fever of other and unknown origin |
| R63 | Symptoms and signs concerning food and fluid intake |
| R59 | Enlarged lymph nodes |
| F11 | Mental and behavioural disorders due to use of opioids |
| F01 | Vascular dementia |

Table S2- Window of contact with secondary healthcare among people diagnosed with blood-borne viruses in Scotland, by late diagnosis, 2018-2022I

|  | **HIV** | | | | **HCV** | | | | **HBV** | | | |
| --- | --- | --- | --- | --- | --- | --- | --- | --- | --- | --- | --- | --- |
|  | **Total (col%)** | **Late diagnosis** | | **p-value** | **Total (col%)** | **Late diagnosis** | | **p-value** | **Total (col%)** | **Late diagnosis** | | **p-value** |
|  |  | **Yes (col%)** | **No (col%)** |  |  | **Yes (col%)** | **No (col%)** |  |  | **Yes (col%)** | **No (col%)** |  |
| **Total number of diagnoses** | 543 | 204 | 339 |  | 4,379 | 99 | 4,280 | - | 1,608 | 34 | 1,574 | - |
| **Secondary care** | | | | | | | | | | | | |
| **Admitted to hospital >2 months and <5 years prior to diagnosis** |  |  |  |  |  |  |  |  |  |  |  |  |
| Yes | 137 (25.2%) | 55 (27.0%) | 82 (24.2%) |  | 2,091 (47.8%) | 46 (46.5%) | 2,045 (47.8%) |  | 240 (14.9%) | 13 (38.2%) | 227 (14.4%) |  |
| No | 406 (74.8%) | 149 (73.0%) | 257 (75.8%) | 0.534 | 2,288 (52.2%) | 53 (53.5%) | 2,235 (52.2%) | 0.875 | 1,368 (85.1%) | 21 (61.8%) | 1,347 (85.6%) | < 0.001 |
| **Admitted to hospital >2 months and <2 years prior to diagnosis** |  |  |  |  |  |  |  |  |  |  |  |  |
| Yes | 101 (18.6%) | 40 (19.6%) | 61 (18.0%) |  | 1,446 (33.0%) | 29 (29.3%) | 1,417 (33.1%) |  | 171 (10.6%) | 8 (23.5%) | 163 (10.4%) |  |
| No | 442 (81.4%) | 164 (80.4%) | 278 (82.0%) | 0.732 | 2,933 (67.0%) | 70 (70.7%) | 2,863 (66.9%) | 0.490 | 1,437 (89.4%) | 26 (76.5%) | 1,411 (89.6%) | 0.029 |
| **Emergency departments (ED)** | | | | | | | | | | | | |
| **Attended ED >2 months and <5 years prior to diagnosis** |  |  |  |  |  |  |  |  |  |  |  |  |
| Yes | 215 (39.6%) | 90 (44.1%) | 125 (36.9%) |  | 2,848 (65.0%) | 60 (60.6%) | 2,788 (65.1%) |  | 373 (23.2%) | 17 (50.0%) | 356 (22.6%) |  |
| No | 328 (60.4%) | 114 (55.9%) | 214 (63.1%) | 0.113 | 1,531 (35.0%) | 39 (39.4%) | 1,492 (34.9%) | 0.407 | 1,235 (76.8%) | 17 (50.0%) | 1,218 (77.4%) | < 0.001 |
| **Attended ED >2 months and <2 years prior to diagnosis** |  |  |  |  |  |  |  |  |  |  |  |  |
| Yes | 149 (27.4%) | 66 (32.4%) | 83 (24.5%) |  | 2,178 (49.7%) | 43 (43.4%) | 2,135 (49.9%) |  | 267 (16.6%) | 13 (38.2%) | 254 (16.1%) |  |
| No | 394 (72.6%) | 138 (67.6%) | 256 (75.5%) | 0.058 | 2,201 (50.3%) | 56 (56.6%) | 2,145 (50.1%) | 0.243 | 1,341 (83.4%) | 21 (61.8%) | 1,320 (83.9%) | 0.001 |

**Table S3 - Associations between attending an emergency department in the period between 2 years and 2 months prior to diagnosis and late diagnosis for individuals newly diagnosed with BBVs in Scotland, 2018-2022.**

|  | **N** | **Late diagnosis (% of N)** | **Uni-variate** | | **Multi-variate ^a^** | |
| --- | --- | --- | --- | --- | --- | --- |
|  |  |  | **OR (95% CI)** | ***p*-value** | **aOR (95% CI)** | ***p*-value** |
| **HIV** | | | | | | |
| **ED attendance ^a,b^** |  |  |  |  |  |  |
| No | 394 | 138 (35.0%) | 1 |  | 1 |  |
| Yes | 149 | 66 (44.3%) | 1.48 (1.00 to 2.16) | 0.047 | 1.54 (1.04 to 2.27) | 0.029 |
| **HCV** | | | | | | |
| **ED attendance ^a,b^** |  |  |  |  |  |  |
| No | 2,172 | 56 (2.6%) | 1 |  | 1 |  |
| Yes | 2,178 | 43 (2.0%) | 0.76 (0.51,1.14) | 0.183 | 1.28 (0.83,1.96) | 0.262 |
| **HBV** | | | | | | |
| **ED attendance ^a,b^** |  |  |  |  |  |  |
| No | 1,337 | 21 (1.6%) | 1 |  | 1 |  |
| Yes | 267 | 13 (4.9%) | 3.23 (1.55, 6.41) | 0.001 | 1.54 (0.69, 3.29) | 0.277 |

^b^Adjusted for age and sex

**Table S4 - Associations between admission to hospital in the period between 2 years and 2 months prior to diagnosis and late diagnosis for individuals newly diagnosed with BBVs in Scotland, 2018-2022.**

|  | **N** | **Late diagnosis (% of N)** | **Uni-variate** | | **Multi-variate ^a^** | |
| --- | --- | --- | --- | --- | --- | --- |
|  |  |  | **OR (95% CI)** | ***p*-value** | **aOR (95% CI)** | ***p*-value** |
| **HIV** | | | | | | |
| **Admitted to hospital ^a,b^** |  |  |  |  |  |  |
| No | 442 | 164 (37.1%) | 1 |  | 1 |  |
| Yes | 101 | 40 (39.6%) | 1.11 (0.71 to 1.73) | 0.640 | 1.08 (0.69 to 1.68) | 0.332 |
| **HCV** | | | | | | |
| **Admitted to hospital ^a,b^** |  |  |  |  |  |  |
| No | 2,904 | 70 (2.4%) | 1 |  | 1 |  |
| Yes | 1,446 | 29 (2.0%) | 0.83 (0.53, 1.27) | 0.4 | 1 (0.63, 1.55) | 0.994 |
| **HBV** | | | | | | |
| **Admitted to hospital ^a,b^** |  |  |  |  |  |  |
| No | 1,433 | 26 (1.81%) | 1 |  | 1 |  |
| Yes | 171 | 8 (4.68%) | 2.68 (1.11, 5.71) | 0.018 | 0.63 (0.23, 1.60) | 0.354 |

^b^Adjusted for age and sex
